# Neuropathic pain: long term follow-up

**DOI:** 10.1186/2050-5736-3-S1-O22

**Published:** 2015-06-30

**Authors:** Ernst Martin, Beat Werner, Ronald Bauer

**Affiliations:** 1 University Children’s Hospital Zurich, Zurich, Switzerland; 2 Kantonsspital St. Gallen, St. Gallen, Switzerland

## Background/introduction

Radio frequency induced thalamotomy has been used in patients with neuropathic pain for many years. Today, transcranial MR imaging-guided focused ultrasound (tcMRIgFUS) offers the opportunity to conduct this treatment non-invasively under closed-loop MR-imaging guidance.

## Methods

So far we have treated a total of 23 patients suffering from chronic neuropathic pain of various origins using tcMRIgFUS. Targeting the posterior part of the central lateral nucleus of the thalamus (pCL), we applied both unilateral and bilateral lesions, depending on the severity and extent of the clinical symptoms. Also, depending on clinical presentation, the effected lesions were created either by a single ablation cell, or, where the lesion volume of a single ablation cell seemed insufficient, by combining two to three adjoining ablation cells that were generated by electronically steering the acoustic focus a few millimetres in order to increase the lesion volume. Using a custom built eight channel phased array coil which snugly fits around the ultrasound transducer, high resolution images could be acquired during the whole intervention procedure. The temperature evolution in the target region was continuously monitored by MR-thermometry and therapeutically effective temperatures were considered to lie between 54 and 62°C.

## Results and conclusions

Long term effects resulting from pCL thalamotomy with tcMRIgFUS in the 23 patients evaluated here during a follow-up period of 1 to 2 years will be presented. The median pain relief is 56%, with an average reduction of subjectively felt maximal pain intensity of 34% estimated by the patients on a numerical rating scale. Here we will present important physical parameters such as lesion size and its evolution over time, as well as maximal temperature achieved during the FUS intervention. We will demonstrate the relationship between therapeutic effect and lesion size and Tmax, respectively. Apart from the pain relief, changes in quality of life and activity of daily living are at least as important for the patients. The results will also demonstrate the mutual compliment of deep brain stimulation and focused ultrasound. They form the basis for discussing possible improvements in patient management and new methodological approaches in order to allow application of tcMRIgFUS also in difficult and desperate patient situations.

